# Effects of Engineered Nanomaterials on Plants Growth: An Overview

**DOI:** 10.1155/2014/641759

**Published:** 2014-08-14

**Authors:** Farzad Aslani, Samira Bagheri, Nurhidayatullaili Muhd Julkapli, Abdul Shukor Juraimi, Farahnaz Sadat Golestan Hashemi, Ali Baghdadi

**Affiliations:** ^1^Department of Crop Science, Faculty of Agriculture, Universiti Putra Malaysia (UPM), 43400 Serdang, Selangor, Malaysia; ^2^Nanotechnology and Catalysis Research Centre (NANOCAT), University Malaya, IPS Building, 50603 Kuala Lumpur, Malaysia; ^3^Institute of Tropical Agriculture, Universiti Putra Malaysia (UPM), 43400 Serdang, Selangor, Malaysia

## Abstract

Rapid development and wide applications of nanotechnology brought about a significant increment on the number of engineered nanomaterials (ENs) inevitably entering our living system. Plants comprise of a very important living component of the terrestrial ecosystem. Studies on the influence of engineered nanomaterials (carbon and metal/metal oxides based) on plant growth indicated that in the excess content, engineered nanomaterials influences seed germination. It assessed the shoot-to-root ratio and the growth of the seedlings. From the toxicological studies to date, certain types of engineered nanomaterials can be toxic once they are not bound to a substrate or if they are freely circulating in living systems. It is assumed that the different types of engineered nanomaterials affect the different routes, behavior, and the capability of the plants. Furthermore, different, or even opposing conclusions, have been drawn from most studies on the interactions between engineered nanomaterials with plants. Therefore, this paper comprehensively reviews the studies on the different types of engineered nanomaterials and their interactions with different plant species, including the phytotoxicity, uptakes, and translocation of engineered nanomaterials by the plant at the whole plant and cellular level.

## 1. Nanotechnology: In General

The nanotechnology process began with the generation, manipulation, and deployment of nanomaterials, representing an area holding significant promise for a wide range of applications [1–5]. Nanotechnology has become a dynamically developing industry, with multiple applications in energy, materials, computer chips, manufacturing, health care, and medical diagnosis [2, 3]. Products that are derived from nanotechnology are known as nanomaterials [4]. It is believed that there are over 800 nanomaterial products currently available in the market, and it is expected to increase over the next few years [5–7]. Thus, through 2014, it also approximated that an excess of 15% of all products on the worldwide market would have some type of nanotechnology integrated within their production processes [6].

### 1.1. Nanomaterials

Generally, nanomaterials refer to a colloidal particulate system, with size ranging from 10 to 1000 nm, possessing unique properties, such as size-dependent qualities, high surface-to-volume ratio, and promising optical properties [4, 5]. The main categories of nanomaterials are carbonaceous [8], semiconductor, metal oxides [9, 10], lipids [11], zero-valent metals [12], quantum dots, nanopolymer [13], and dendimers [14], with different kinds of features, such as nanofibers, nanowires, and nanosheets (Table 1). The preparation of nanomaterial typically involves a direct and synthetic route that yields particles in the nanosize range, followed by the application of grinding or milling, high pressure homogenization, and sonication to reduce its size [15, 16]. Meanwhile, the bottom-up process in synthesizing nanomaterials involved reactive precipitation and solvent displacements [17].

It is very important to realize that nanomaterials, due to their enhanced contact surface area, might be poisonous, an effect which might be absent in its bulk counterpart, especially in an open agricultural ecosystem [9, 10]. Examples of these cases are solid matrices with nanomaterials that have a nanostructure freely attached to its surface, where it can moderately be expected to break free or leach out once coming into contact with water or air, or when subjected to reasonably foreseeable mechanical forces [14].

### 1.2. Engineered Nanomaterials

**Figure 1:**
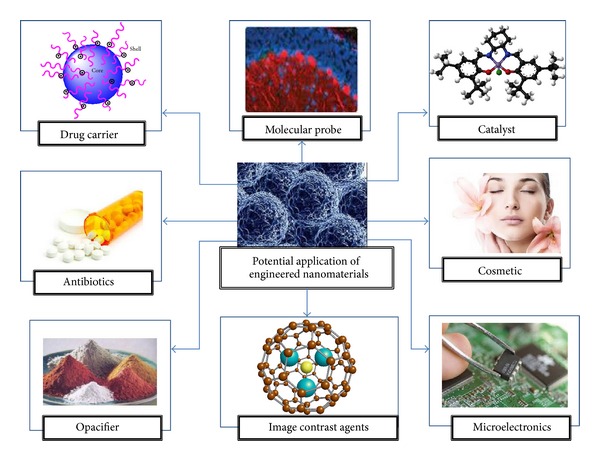
List of carbon-based nanomaterials potential applications.

More than 1300 commercial nanomaterials, with widespread of potential applications, are currently available [15–17, 19]. Carbon nanotubes and related materials were discovered in 1985 [8]. By 2011, the annual worldwide production of carbon-based nanomaterials was estimated to exceed 1000 tons, with some of the factory's capacity reaching to 1500 tons per year [20–22]. The first product was shown to be multiwall carbon nanotubes, with concentric cylinders reaching to 10 *μ*m in length and 5–40 nm in diameter [21]. Consequently, a single walled carbon nanotube (SWCNTs) has been synthesized with the assistance of Co/Ni catalyst [23]. This fullerene structure exhibited promising electrical/thermal conductivity and mechanical properties. For example, a single walled carbon nanotube has a strength-to-weight ratio that is 460 times stronger than that of steel [23, 24]. The behavior of carbon-based nanomaterials is reflective of different environments and conditions [22]. For example, once the carbon-based nanomaterials have been introduced to the human health area, it will group itself with other tubes and rods as high aspect ratio nanomaterials, similar to asbestos [25]. Meanwhile, due to its inherent hydrophilicity, carbon-based nanomaterials tend to precipitate and aggregate in aqueous mediums [21]. Some studies have focused on the surface functionalization of carbon-based nanomaterials, such as the attachment of polyethylene glycol, noncovalent modification, self-assembly, and conjugation of phospholipids, lysophosphtidylcholide, and lysophosphatidylcholine to increase its stability, especially in aqueous suspension [26, 27]. This, in return, increases the application range of carbon-based nanomaterials and its derivatives in catalyst, fuel cell electrodes, orthopedic implants, plastics, battery, super capacitors, water purification system, conductive coatings, adhesive, sensors electronics, composites, aircraft, and automotive industries (Figure 1).

It has been documented that carbon-based nanomaterials such as nanotubes and fullerenes could be degraded under a wide range of conditions, whereby fullerene has a tendency to be taken up by wood decay fungi and metabolized [21–27]. The fullerene nanoparticles would accumulate in microbial cells, followed by eating mechanism of worms, thus increasing the possibility of nanomaterials to be incorporated into the food chain [24].

The next class of engineered nanomaterials is metal-containing materials, such as metal oxides [9]. The synthesis of metal oxides and metal nanoparticles could be achieved via several routes. Grinding of bulk materials is the usual practice for synthesizing metal oxide nanoparticles [9, 32]. The range of nanoparticulate metal oxides includes both individual (e.g., CeO_2_, TiO_2_, ZnO, CrO_2_, MoO_3_, and Bi_2_O_3_) and binary oxides (e.g., BaTiO_2_, LiCoO_2_, and InSnO). This series of metal oxide found a wide industrial application. For example, owing to its ultraviolet blocking ability and visible transparency of nanoparticle foam, ZnO and TiO_2_ are extensively being used in cosmetics, sunscreen, and bottle coatings [33]. It was reported that in 2005–2010, the production of ZnO and TiO_2_ for the application of skin care products reached to 1000 tons per year [34]. Moreover, CeO_2_ is finding major utilization as a combustion catalyst in diesel fuels to enhance emission quality, as well as in oxygen pumps, gas sensor, solar cells, and metallurgical ceramic/gas applications [35].

## 2. Engineered Nanomaterials: In Living System

**Figure 2:**
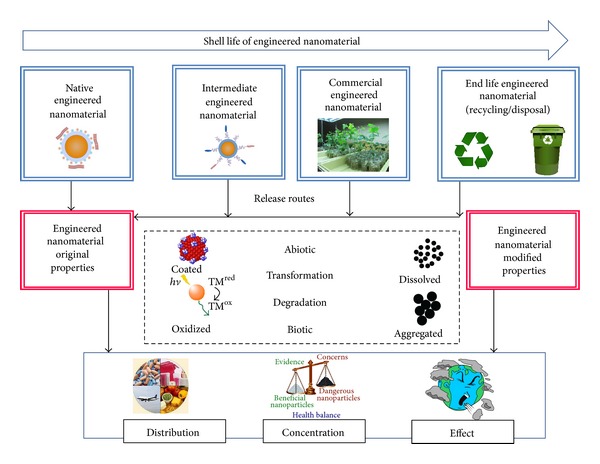
Release routes of engineered nanomaterials in living system.

The advent of nanomaterials has seen increased production recently, and its interaction with living organisms is a significant cause of concern [10–14, 36]. Manufactured ENs enter living systems through intentional and unintentional releases such as solid/liquid waste streams from manufacture facilities and atmospheric emissions [4]. Nanomaterials can come into contact with living organisms via multiple routes (Figure 2), such as incidental release, direct release from industrial products or processes, as well as commercial products during intended uses that in turn enter the sewer-to-wastewater treatment plants [37, 38].

Its application continues in biosolids from wastewater treatment fields, pesticides applied to agricultural, paints, fabrics, personal health care, and accidental spill of materials during manufacturing, contact during usage of consumer products, and direct infiltration or runoff of excretion from humans or livestock [39]. For example, ENs have been applied to remediate groundwater, where its filtration from stack emissions needs a new generation of nanostructured sorbents for an effective removal [40–42]. Compared to the nanomaterials from diesel emission, the emitted nanomaterials from wastewater would eventually be deposited on the surface water bodies and land despite the fact that the treatment for avoiding aggregation may lead to its buoyancy's increment. Once the nanomaterials reach land, they have the potential to pollute soil, migrate into surface/ground water, and interact with biota. This nanoparticle can also be transported to an aquatic system by rainwater and/or wind runoff. Upon release to water, dispersed nanomaterials are anticipated to behave based on the phenomenon of colloidal science [39]. Generally, colloidal suspensions of ENs are generally unstable, where particles may approach each other close enough for attractive Van der Waals force to become dominant over repulsive electrostatic forces and steric hindrance [41, 42]. This resulted in particles adhering to each other, followed by sedimentation [40]. Furthermore, natural waters contain other adherent matters such as solid, dissolved, or colloidal materials. In addition, the suspensions of dispersed nanomaterials are stable only under narrow ranges of environmental conditions, where ionic strength, pH, and the presence of natural organic matter should be taken into account [37, 38]. For example, seawater has a high pH and ionic strength, thus, electric double layers of colloid particles are smaller compared to freshwater [39, 43]. This in turn allowed closer interparticle approach, leading to higher aggregations.

Since the last decade, an intensive wealth of acute toxicity studies focusing on biological and ecological effect on short-term effect of ENs has garnered interest. However, there is still a gap of research interest in the effect on the presence of ENs in living environments. For example, studies on the estimation of appropriate exposure are hampered by the deficiency of knowledge of rates of release or concentrations of nanomaterials in the environment [44]. There is also a lack of knowledge related to the theory of estimating environmental concentrations focusing on the releasing rate [45, 46]. It has been reported that the existing theory of behavior of chemicals and particulates in the environment is not aligned with the characteristics of nanomaterials. Recently, the theory is only based on the direct measurement and assessment on the existing nanomaterials in living systems, focusing much more on the parameters involved (e.g., acidity, pH, charged ions, and level of organic matter) for the release mechanism [42]. Those theories reported two main conclusions; first, the quantity estimation of nanomaterials in water surface is in accordance to the prediction rather than the actual measurement. Second, nanomaterials could be dissolved, latched onto chemical molecules/ions, clamped, transformed into other chemicals by microorganism, or undergone a mineralization before being 100% degraded with the dissolution of organic carbon or the generation of CO_2_ [47–49]. There are some reports that claimed that whether or not it is followed by degradation of the dissolved material, the process of dissolution makes nanoparticles disappear and become less persistent. In principle, the methods measuring CO_2_ production require a larger amount of test materials [48].

With that in mind, studies that are more extensive need to focus on the possibility of the potential places where ENs are concentrated, agglomerated, or interacted with organic matters [49]. This is important, especially in cases of wastewater treatment, where it will likely be sites for the accumulation of some ENs in sewage. There is also the possibility of bioaccumulations through the concentration of ENs in particular organs [39, 50]. Through the bioaccumulation process, the aggregation of ENs will end up with sedimentation, where the organism may take up ENs via inhalation or ingestion. Some may also transfer the microorganism across epithelial surfaces (e.g., lining of the lungs, gills, skin, or intestines). Meanwhile, microorganism can also take up ENs via simple diffusion transport across cell membranes, or even after membrane damage [51]. In this case, the transfer mechanism relies on the dispersion, concentration, and dissolving of ENs before ingestion. In addition, some semiconductor-engineered nanomaterials could also be concentrated by the waters, which could be directly transferred to the ecosystems' food chains [52–54]. For example, a recent study about the achievable transfer of quantum dots in a simple aquatic food chain has been conducted, and it was reported that nanomaterials could be transferred to rotifers via dietary uptake of ciliated protozoans. Other studies claimed that there is some potential for transferring nanomaterials across food chain levels, depending on the material type and food chain [54, 55].

This creates problems for aquatic microorganism, since nanomaterials themselves have an antibacterial or virucidal effect, especially in cases such as silver nanoparticles [56]. For example, studies related to water and land dwelling organisms have shown a wide range of effects on the presence of different ENs on microorganism, invertebrates, and fish. The results so far pointed out the potential for hazardous effects at lethal and sublethal levels (e.g., behavior, reproduction, growth, and development) towards the production of reactive O_2_ species, inflammatory responses, and cytostatic effects [57–59]. This is supported by some of the studies on the potential effects on the movement of ENs to embryos, accumulation, and food chain transfer [58].

With that in mind, some issues should be addressed and highlighted concerning the presence of ENs in the living system. The issues ranged from the behavior of nanomaterials manufacturers in the environment, the stability of ENs, effect on aquatic/sedimentary biota not being similar to nonnanomaterials of the same materials, and suitable protection of the ecosystem while permitting the advantages that nanotechnology offers to be fully developed [58–61]. This started with the knowledge of colloidal science, which could provide evidence of the physical and chemical characters of nanomaterials in the receiving environment [48–51]. Generally, small particles tend to agglomerate or aggregate relative to other colloidal, which accounts for its particulate being present in the environment [34]. In the context of the environment, ENs may be present and cannot be disassociated by either dissolution or agglomeration [44]. As far as we can tell, there are no peer-reviewed publications on the concentration of ENs in environmental compartments, such as surface water or soil [35, 37–41]. Indeed, the quantity estimation of ENs is based on its predicted rather than the actual values, which could be considered suitable metrics of the accurate measurement of ENs in living system risk assessment not being finalized and still under discussion [32]. Several considerations should be taken into account in the assessment effect of nanomaterials in living systems, where the exposure concentration/doses should be realistic. In this case, the assessment should not only cover the free nanoparticle form, but also all physical and chemical species, aggregated matter, and associated/deposited matter with other organic compounds. This would have influenced ENs bioavailability, which will in turn determine the biological uptakes.

### 2.1. Engineered Nanomaterials in Agriculture

**Figure 3:**
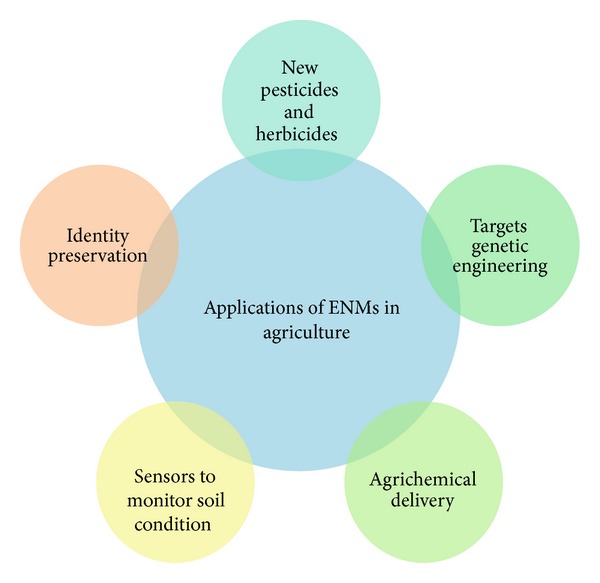
General application of engineered nanomaterials in agricultural.

Engineered nanomaterials research and development, in agricultural applications, probably facilitated and framed the next stage of development of genetically modified crops (GMCs), animal production input, biocides, and precision farming system [59–64]. Similar to other technologies, low-cost ENs and field application technologies are required for their applications in agriculture [1–5]. Nanotechnology is the result of the improvement associated with a variety of economical applications for superior plant growth. Applications of ENs motivate earlier plant germination as well as enhance plant production (Figure 3).

Nanoagriculture utilizes nanotechnology to improve the yield of plants for food, fuel, and other uses. Researchers report a big gap in knowledge about the effects of nanoparticles on rice, tomatoes, corn, and other food crops [63–65]. The build-up and uptake of ENs differs and these kinds of components mainly rely on the type of plant, the chemical composition, and the size of ENs [66–68]. Some plants are capable of uptaking and accumulating engineered nanomaterials. The interaction of plant cell with the ENs leads to the modification of plant gene expression and associated biological pathways, which eventually affect plant growth and developments [67–69]. The effects of ENs on different plant species can vary greatly with plant growth stages, method, and duration of exposure and depend on the ENs shape, size, chemical composition, concentration, surface structure, aggregation, and solubility [68].

To our knowledge, the first report relevant to the effects of bulk and ENs on Sage (*Salvia officinalis L.*) is in [69]. Nanomaterials improved seed germination in plants but can have contrary effects on others [63, 64]. In these instances, studies on ENs aspects are on the nanoparticles dosage in the various mediums, their chemical and physical properties, the mechanisms permitting them to pass through cellular membranes and cell walls. The specific properties, which might be in connection with positive and negative effects of nanoparticles and the mechanism underlying nanoparticles trophic transports are necessary as well [65]. Moreover, the existing application methods need to be reviewed for improved efficiency of nanomaterials on future targets. The necessity of further studies on the possible risks related to the use of nanomaterials and their potential adverse effects is needed [60–62].

Research indicates that extended amounts of ENs are highly toxic to aquatic life, bacteria, and human cells in vitro. At the nanoscale, even normally benign substances may become hazardous. According to the particle physics and studies of fine atmospheric contaminants, ENs are usually in the size range that stays suspended for days to weeks if released into the air [54–56]. ENs are inhaled and collected in all regions of the respiratory system of the plant. Because ENs is small, they follow airstreams more easily than larger particles are simply collected and taken in standard ventilated enclosures [5–9]. Therefore, a particular concern is the ability of the nanoparticle directly taken up by individual cells and cell nuclei, especially through the respiratory system. Bioaccumulation is another topic of concern [10–12, 36]. As the properties of materials at the nanoscale variable are poorly understood, it is not possible to provide a generic assessment of health and environmental risks [53–55].

Therefore, the interaction associated with plant cell and ENs led to the modification of plant gene expression. It connected biological pathways, which eventually affect the plant growth and development. This is due to the unique properties of ENs that can modify their physicochemical properties and give different effects on plant growth, depending on nanomaterials surface structure, size, shape, chemical composition, concentration, solubility, and aggregation [63]. Hence, ENs ought to be designed to have all necessary properties such as effective concentration with high effectiveness, stability, and solubility, time-controlled release in response to certain stimuli, enhanced targeted activity, and less toxicity with safe and easy mode of delivery to avoid repeated applications [67, 69]. The vast majority of research works done for each different nanomaterials and product in agriculture is thereof to investigate its potential toxicity before its use could consider safe.

## 3. Engineered Nanomaterials: On Plant Growth

**Figure 4:**
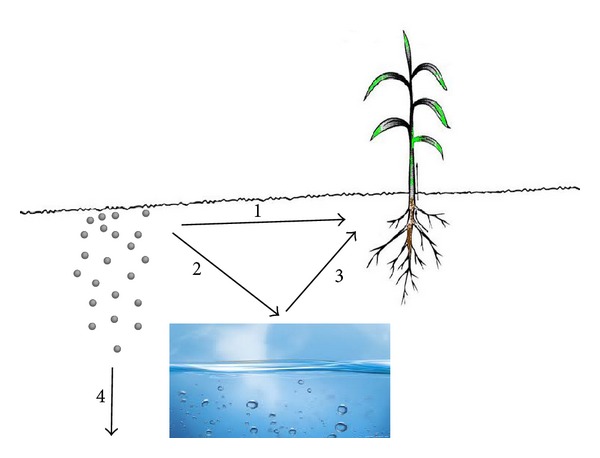
Interaction of engineered nanomaterials in the environment. (1) Engineered nanomaterials absorbed directly to plant root. (2) Engineered nanomaterials mixed with water medium. (3) Engineered nanomaterials mixed with water and transferred to plant. (4) Engineered nanomaterials stayed in the soil.

The possibility of plants interacting with ENs is increased with the application of its production and application in the variety of instruments and goods. Underneath particular growing environments, plants may possibly absorb essential and nonessential elements, which to certain concentration, might result in toxicity [70–72]. It has been documented that, toxic elements with no known function in biological systems usually accumulate in plant tissues and cause some lethal effect for nontolerant species [55, 71]. ENs can reach plants through direct application, accidental release, contaminated soil/sediments, or atmospheric fallouts, which results in a significant negative effect on food crops and food chains (Figure 4).

It is worse when these toxic elements are transferred from plants to consumers. Research studies on selenium have found that selenium-laden plants can be used to supply selenium deficiencies in ruminants and other animals, even at very narrow and low deficiencies and toxicities [63, 72]. This has been supported by other studies that focused on the toxicity of various types of plants, including radish (*Raphanus sativus*), corn (*Zea mays*), lettuce (*Lactuca sativa*), cucumber (*Cucumis sativus*), rape (*Brassica napus*), and many more [69, 70]. However, the biodegradation screening method of measuring dissolved on toxicity of both carbon and metal-based nanomaterials is inapplicable. Indeed, most studies concentrated on the uptake, accumulation, translocation, and biotransformation of ENs.

### 3.1. Carbon Based Engineered Nanomaterials: On Plant Growth

A wide production of carbon-based nanomaterials has led to its potential release in living systems, either intentionally in discharges, or unintentionally in spillages, and greater possibilities of the adverse environmental effects [8]. Among carbon-based nanomaterials, the most studied materials are fullerene C_70_, fullerol (C_60_(OH)_20_), and carbon nanotubes. As carbon-based nanomaterials are considered highly hydrophobic with the tendency to aggregate, it could be expected to settle in the living system [73]. This hydrophobic property would enhance the carbon-based nanomaterials' capability to interact with many organic substances. Thus, the only low surface friction of carbon nanotubes is required to assist the flow of organic substances into the cytoplasm [74]. Some edible plants can take up some carbon-based nanoparticle, with specific uptake mechanism and accumulation. Properly functionalized ENs provided better penetration through the cuticle. This in turn allows for a slow and governed discharge of active ingredients on reaching the target weed. For instance, appropriately functionalized lipophilic nanosilica gets absorbed into the cuticular lipids of insects by physisorption, damages the protective wax layer, and induces death by desiccation.

#### 3.1.1. Fullerene: On Plant Growth

It has been reported that the presence of fullerene in the form of black aggregates is more plentiful in seeds and roots compared to the leaves and stems for rice seeds [73–75]. However, in mature plants, more robust translocation from the roots of the aerial part of the plant is observed. Thus, fullerene aggregates were mostly present in or near the stems vascular system and leaves, whereby the roots have been devoid of fullerene [74]. The aggregation of fullerene in leaves indicated that they followed the transmission route of nutrients and water through the xylem [76]. It is believed that the individual fullerenes nanoparticle entering the plant roots through osmotic pressure, capillary forces, and pores in the cell walls by the intercellular plasmodesmata, or by means of the greatly regulated symplastic routes [77, 78]. Only the fullerene particles with a diameter of less than the pore diameter of the cell wall could simply pass through and reach the plasma membrane [74].

#### 3.1.2. Fullerol: On Plant Growth

The small size and hydrophobicity properties inducing a permeability of fullerol through the cell wall pores in the plant cell suspension leads to minimal uptake of the nanoparticles [79]. Consequently, the fullerol have accumulated at the interface between the cell wall and plasma membrane [77]. This accumulation also occurred between adjacent epidermal cell walls, showing its apoplectic mode of transport in the plant tissues [77, 79].

#### 3.1.3. Carbon Nanotubes: On Plant Growth

Carbon nanotubes (CNTs) may have possibly single or multiple layers of carbons established in a cylinder [80–84]. CNTs behave as fibers, with its properties very different from bulk carbon or graphite [81]. Thus, CNTs possess excellent tensile strength and are possibly the strongest, smallest fiber known [82–84]. Most studies are increasingly carried out in order to obtain the uptake and transport mechanism of carbon-based nanomaterials into intact plant cells [85–87]. There is proof that CNTs could translocate to systemic sites, such as fruits, leaves and roots, which could involve a strong interaction with the cells of the tomato seedling. This resulted in significant changes in total fruits, leaves, and roots gene expression [86]. CNTs have phytotoxic effects on plant cells due to aggregation and causes cell death in a dose dependent manner [73]. Cell death is demonstrated by electrolyte leakage and the swelling of the cell plant.

Theoretically, single wall carbon nanotube is too large to penetrate the cell wall. However, the evidence of an endocytosis-like structure of the plasma membrane in an* Arabidopsis thaliana* leaf cell indicates the existence carbon nanotubes and is extremely relevant to guide for additional studies with other edible plants [88, 89]. Then, researches with cell suspensions of* Nicotiana tabacum* cv. Bright Yellow found that the water-soluble single wall carbon nanotube with a length of less than 500 nm has penetrated the intact cell wall and membrane over fluidic phase endocytosis [90, 91]. Due to its small size, carbon nanotubes tend to interact with the polysaccharides and proteins in the cell wall and elicit hypersensitive retorts mimicking plant pathogens, leading to cell mortality [80–83]. This is supported by the recognition of noncovalent interactions involving rice mortality and carbon nanotubes. Thus, CNTs could possibly improve root growth of cucumbers (*Cucumis sativus*), onions (*Allium cepa*), and nanotube sheets formed by both functionalized-CNTs and nonfunctionalized CNTs on root surfaces, but none entered the roots [92, 93]. Though CNTs were discovered to diminish root growth in tomato plants, recent works reported that CNTs penetrated tomato seed coat and significantly enhance seedling growth and seed germination rates [93–95].

#### 3.1.4. MWCNTs: On Plant Growth

Multiwalled carbon nanotubes (MWCNTs) are 1 mm long and 20 nm in diameter [96–99]. MWCNTs are taken up by the seeds and roots system via the creation of new pores and water uptake in order to develop tomato seedlings [100, 101]. In this case, MWCNTs are visualized to be on the root surface before eventually piercing the epidermal and root hair cell walls and cap of the seedlings [102]. Furthermore, some studies described that MWCNTs permeate tomato seeds and boost the germination rate by improving the seed water uptake [101]. The MWCNTs elevated the germination of seed to up to 90% in 20 days compared to 71% in the control sample and the plants' biomass [103]. Other researches pointed out that the cell walls of rice cell suspension restrict the entry of the MWCNTs into the cellular cytoplasm, forming black clumps that strongly wrap around and associate with the cells [100]. The presence of the clumps, with an increase in the concentration of carbon nanomaterials, would increase in both size and number [101]. This hypersensitive response is thought to be in charge of preventing the entry of MWCNTs through the plant cell walls [101–103]. For example, the seeds treated with MWCNTs showed a few aggregate nanotubes in the vascular system, and none in the tissues. Meanwhile, in the zucchini species, there are no negative effects noticed on seed germination and root elongation within the examined range of MWCNTs [104].

#### 3.1.5. SWCNTs: On Plant Growth

The dimension of typical single walled carbon nanotube (SWCNTs) is about 1 to 2 nm in diameter and 0.1 *μ*m in length [24, 105, 106]. Some studies indicate that the surface modifications of carbon-based nanomaterials increased its widespread, dispensability, and water column stability [107–111]. Contrarily, no uptake of SWCNTs and its functionalized roots of cucumber seedling are found after treatment for 84 h. In the form of nanotube sheet, the SWCNTs were found adhered to the external surface of the main and secondary roots [112, 113]. However, current results are insufficient to determine the translocation of SWCNTs from the root systems to the aerial parts of the plant [110–116].

#### 3.1.6. Graphene: On Plant Growth

**Figure 5:**
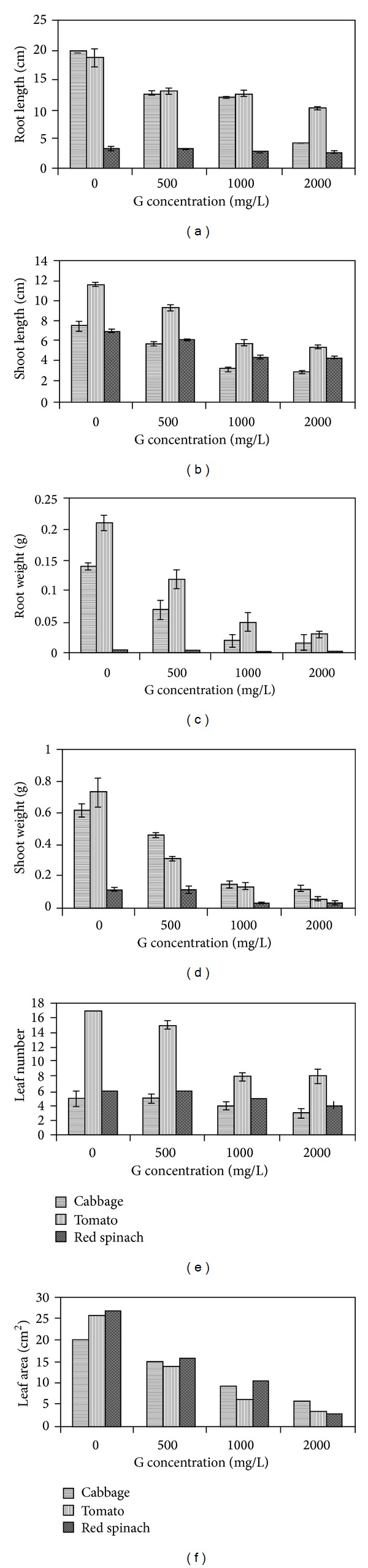
Effect of graphene (G) on of red spinach, cabbage, and tomato seedlings. 21 days seedlings growth on Hoagland media with graphene (0, 500, 1000, and 2000 mgL^−1^) were utilized for all measurements. (a) Root length, (b) shoot length, (c) root weight, (d) shoot weight, (e) leaf number, and (f) leaf area [28].

Graphene is a two dimensional crystalline allotrope of carbon, which can be described as a one atom layer of graphite. At high concentrations of graphene (1000 mgL^−1^), the root hair growth of red spinach and cabbage decreased compared to the control plant [117–119] (Figure 5). This is due to the accumulation of graphene using H_2_O_2_ visualization, together with visible signs of necrotic damage lesions and proof of a massive electrolyte leakage, indicating an oxidation stress mechanism [28, 118, 120].

**Figure 6:**
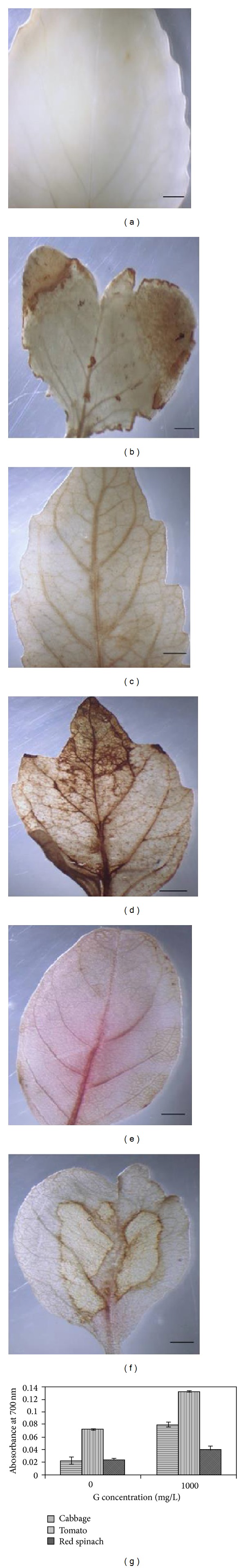
Effects of graphene (G) on accumulation of H_2_O_2_ in leaves tested by means of the ROS-sensitive dye DAB of red spinach, cabbage, and tomato seedlings. 21 days leaves treated with or without 1000 mgL^−1^ graphene were utilized for all measurements. (a), (c), and (e) are cabbage, tomato, and red spinach leaves without graphene, respectively. (b), (d), and (f) are cabbage, tomato, and red spinach leaves with graphene (1000 mgL^−1^), respectively. The brown staining shows the formation of a brown polymerization product when H_2_O_2_ reacts with DAB. (g) Effect of graphene (1000 mgL^−1^) on the accumulation of H_2_O_2_ in treated leaves as measured utilizing DAB [28].

For example, intracellular reduction oxidation system probably has an essential function in the induction of cell death induced by graphene [28] (Figure 6). It described the accumulation graphene as leading to cell death, shown by electrolyte leakage from cells [119]. Via graphene treatment, the root surface area of cabbage significantly improved, and it may be that an excess of graphene resulted in the swelling in* Origanum vulgare* and* Origanum *[117, 118]. Graphene is known as inducing phytotoxic effects in plant cells due to the accumulation mechanism. This causes cell death and the accumulation in a dose-dependent manner [121, 122]. There is certainly proof that graphene could translocate to systemic sites, such as fruits, roots, and leaves, which engage in a strong interaction with the cells of tomato seedlings, leading to substantial modifications in total gene expression in fruits, leaves, and roots and exerting toxic effects [123–126].

**Figure 7:**
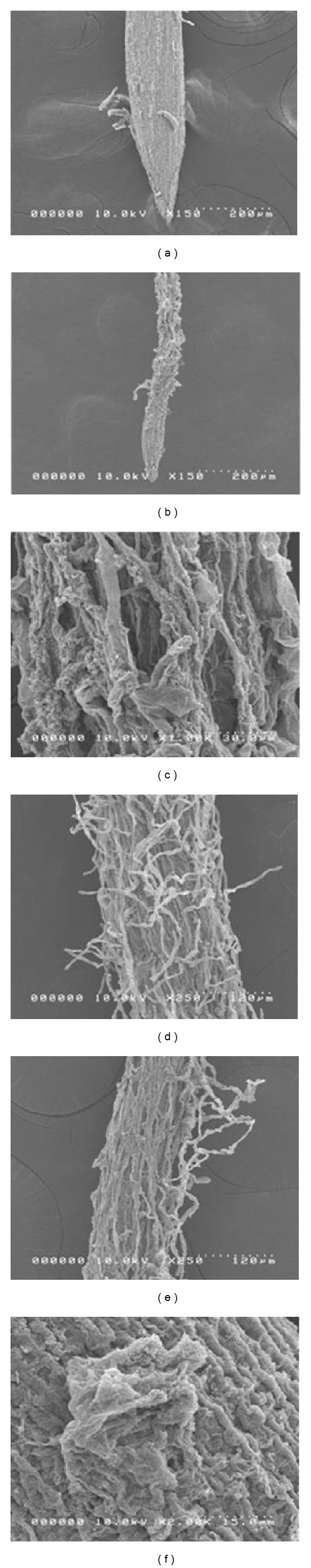
Behavior of graphene (1000 mgL^−1^) on the root surface of tomato seedlings grown in Hoagland medium. (a, d) SEM image of the untreated control of tomato root elongation and root hair zone, respectively. (b) Root elongation zone of tomato root and (c, e, and f) showing surface detachment and aggregates of G on the tomato roots surface treated with G [28].

With that, it is unexpected to find the toxic effects of graphene on terrestrial plant species, in tomato, cabbage, and red spinach [124, 125]. The similar growth pattern observed in tomato, cabbage, and red spinach using graphene nanomaterials was reported in [117, 124, 127]. At higher concentrations of graphene (1000 mgL^−1^), the root hair growth of red spinach and cabbage compared to control plants was reduced [117] (Figure 7).

Overproduction associated with the accumulation induced by graphene could produce substantial plant growth inhibition, and biomass reduction reported that the production of accumulation could be a main factor in the toxicological effects of nanostructured materials [128]. Declaration of accumulation production by means of H_2_O_2_ visualization in addition to visible signs of necrotic damage lesions and evidence of a massive electrolyte leakage all indicated an oxidative stress mechanism mediated through the necrotic pathway, which requires further study [117, 127]. The assessment of graphene toxicity targets terrestrial plant species [125, 126]. It applies a prolonged exposure period with different concentrations to measure potential risks.

### 3.2. Metal and Metal Oxide Nanomaterials: On Plant Growth

Estimates for the production of metal/metal oxide nanoparticles revealed that the quantities produced will probably rise from 2000 tons in 2004, to over 58,000 tons yearly between 2011 and 2020 [129–132]. Metal/metal oxide nanoparticles display size dependent properties, such as fluorescence, photocatalytic degradation, or magnetism, which has biotechnological applications in soil remediation, sensor development, and agrochemicals [130, 131]. In natural living systems, the effect of metal/metal oxide by plants is expected to depend largely on the chemical properties, colloidal properties such as sediments, soil or sludge, and organic content [133, 134]. The most studied metal-based nanomaterials are TiO_2_, CeO_2_, Fe_3_O_4_, and ZnO nanoparticles. Indeed, Fe_3_O_4_ nanoparticle induces some stability effect on aquatic suspensions of fullerene and carbon nanotubes. It has been documented that the effect of humic acids and varying pH can combine the effects on the fate of Fe_3_O_4_ nanoparticle by increasing pH, resulting in a higher level of aggregation. A similar effect was recently shown for CeO_2_.

Metal nanoparticles, under low concentration conditions, play a key role at the limit of plant tolerance in the development of plants [132]. If plants absorb an excess of metals, toxic effects can obvious, including the decrease of growth and irregularities in cell division [129–131]. In this case, excess metal nanoparticles, acting as cofactor for enzymes, are involved in the formation of intermediate metabolites. However, the response of plants to metal nanoparticle varies with the nature of the metal, type of plant species, and the stage of growth.

#### 3.2.1. Au: On Plant Growth

Gold (Au) is categorized as a harmful substance, and the toxicity of Au in many organisms has been reported in ionic or dissolved form [135–138]. The toxicity of Au has been harnessed in the form of Au nanoparticles to act as antibacterial agents in biocide coating, soap, toothpaste and shampoo, and is the most prevalent nanoparticle in over 25 consumer products [136, 137]. The production and the usage of Au nanoparticles in the environment and its potential discharge to the environment might cause severe toxicity problems in the long run [139–141].

For example,* Brassica juncea*,* Medicago sativa* showed an increase in Au uptake, with a conforming improvement in the substrate of Au concentration and exposure time [142–144]. The Au nanoparticles located in the nucleus and the applications of defamation suggested at both species are regarded as hyperaccumulators of Au nanoparticles [145–147]. Additionally, it is documented that Au nanoparticle are transported inside the cells through plasmodesmata. Transmission electron microscope images of rice roots revealed that various Au particle sizes are deposited inside the root cells in the form of vacuoles [143]. The cell damage occurred due to penetrations of large Au nanoparticles entering via small pores [142–145]. Au nanoparticles are reported to disrupt the root tip cells of onion (*Allium cepa*), thus damaging the cell division process by causing the formation of chromatin bridge, cell disintegration, and stickiness [148].

#### 3.2.2. Ag: On Plant Growth

Potential applications of silver (Ag) nanoparticles in biomedicine include imaging applications and chemical sensing. Ag nanoparticles are synthesized using various methods, chemical, electrochemical, photochemical, laser ablations, and others [10]. Although bulk Ag is considered “safe,” Ag nanoparticles need to be examined for environmental impact and biocompatibility if they are to be produced for in vivo usage on a large scale [149–151]. Furthermore, exposure data had shown Ag nanoparticle to be prevalent in the environment, at low but increasing concentrations, with estimation of up to 0.1 and 2.9 mgL^−1^ at the sludge and surface water [152–154]. Thus, some reports suggested the biological effect of Ag nanoparticles might be seen at concentrations of up to 1000 times lower than that for the dissolved Ag^+^ ions [153]. Furthermore, some results from the research proved that the toxicity of Ag nanoparticle is minor at exposures as low as 5 mgL^−1^, with greater inhibitions of growth [152, 154, 155]. It is clear that Ag nanoparticles within the environment pose a potential risk to greater plants, and therefore, the function of ecosystems [153–156]. Ag nanoparticles show adverse effects on seed germinations, root, and shoot growth at concentrations of 4500 *μ*gmL^−1^, 6000 *μ*gmL^−1^, and 3000 *μ*gmL^−1^ on species of rice (*Oryza sativa*), Mung bean (*Vigna radiata*), and Chinese cabbage (*Brassica campestris*), respectively [157–159].

**Figure 8:**
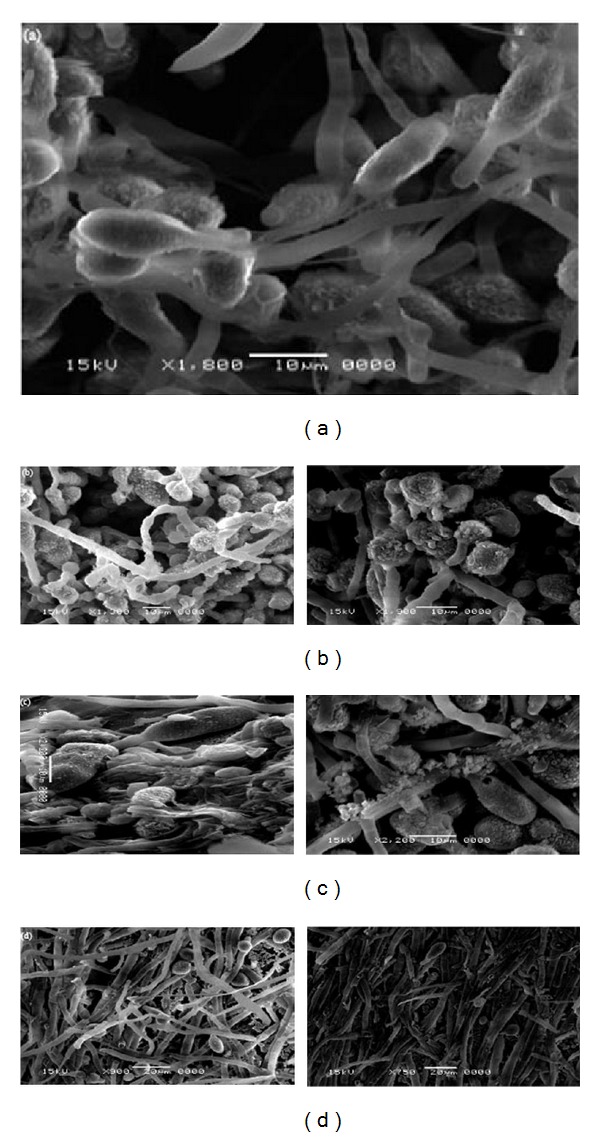
Antifungal effect of Ag nanoparticles on culture filtrate and cell. Scanning electron microscopy images of hyphae of Alternaria alternata treated with silver, copper, or copper/silver nanoparticles. Fungal hyphae grown on potato dextrose agar plates as (a) control or supplemented with 15 mgL^−1^, (b) Ag, (c) Cu, or (d) Ag/Cu nanoparticle solution, respectively, Photos were taken at seven days after the incubation period [29].

Furthermore, Ag nanoparticles with sizes of approximately 40 nm have the potential to cause toxic effects in* Chlamydomonas reinhardtii *algae and* Cucurbita pepo* [160–164]. In the case of* Cucurbita pepo*, Ag nanoparticles induced 4.4 to 10 times more reductions in biomass and transpiration rates, rather than bulk sizes [29, 164–167]. Meanwhile, the limits of uptake and the distribution of Ag nanoparticles have been studied for* Medicago sativa* and* Brassica juncea* species [29, 168–177] (Figure 8).

Various groups have examined the cellular uptake and cytotoxicity of Ag nanoparticles in plant systems. Research on the seed germination and the root growth of zucchini plants in hydroponic solution modified with Ag nanoparticles displayed no negative effects, whereas reductions in plant biomass and transpiration were observed on prolonging the plants' growth in the presence of Ag nanoparticles. The genotoxic and cytotoxic impacts of Ag nanoparticles were studied, utilizing the root tips of onions. Results of Ag nanoparticles impaired the stages of cell division and caused cell disintegration [162, 164]. There are some reports on greater toxic effects in the* Chlamydomonas reinhardtii* algae exposed to Ag nanoparticles as AgNO_3_, at the particle size of 40 nm [164].

There are some studies concentrated on effect of Ag nanoparticle on aquatic plant [168–170]. The studies reported on usage of* Lemna minor L. clone St* to study the phytotoxicity of Ag nanoparticles. The final results demonstrated that the inhibition of plant growth was apparent after exposure to a wide range of Ag nanoparticle (20 to 100 nm), even at low concentrations (5 mgL^−1^) [169–171].

The effects of Ag nanoparticles have also been assessed in many different researches involving plant mediums [178]. The studies focused on soil nematodes, soil microbial community, and other related concerns. It has documented that Ag nanoparticles with sizes of up to 29 nm employed visible reduction effects on the germination of lettuce seeds and cucumber, but no toxic effect has been observed and reported on the reduction germination of barley and ryegrass exposed to Ag nanoparticles. Ag nanoparticles with sizes <100 nm have also shown to reduce the biomass and transpiration of pumpkin (*Cucurbita pepo*) [160]. It reported increased Ag nanoparticles content in the common grass* Lolium multiflorum*, with increasing Ag nanoparticles' concentration. Additionally, the cytological effects of onion (*Allium cepa*) have been reported to include disturbed metaphase, stickiness, chromatin bridge, and other effects. The majority of nanotoxicological researches showed on plants thus far have utilized alternative methods rather than soil media. Most of these researches have been done in an aqueous solution, such as basal medium, deionized water, or Hoagland medium [161].

Few researches documented the toxicity effects of Ag nanoparticles on seed germination, plant uptake, and translocation of nanoparticles in soil [172–174]. Furthermore, the toxicity and bioavailability of Ag nanoparticles to species* Polyboroides radiatus* and* Sorghum bicolor* were measured in both soil medium and agar [175–177, 179].* Polyboroides radiatus* and* Sorghum bicolor* in agar media displayed Ag nanoparticles' concentration dependent-growth inhibition and the EC50s values of* Polyboroides radiatus* and* Sorghum bicolor* calculated to be 13 and 26 mgL^−1^, respectively [178, 180].* Polyboroides radiatus* were not affected by the impediment within the test concentration in the soil media.* S. bicolor* showed a slightly reduced growth rate [178]. Bioavailability and effect of Ag-ions dissolved from Ag nanoparticles are noted to be less in soil than in agar. The results of this research confirmed that bioaccumulation, phytotoxicity, and dissolution of Ag nanoparticles are clearly influenced by the exposure medium [180].

All such studies throw light on the need for a more genotoxic and cytotoxic evaluation by considering the properties of Ag nanoparticles, uptake, translocation, and distribution in different plant tissues.

#### 3.2.3. Cd: On Plant Growth

The short-term effects of Cadmium (Cd) nanoparticles for the root growth of carrot, cucumber, tomato, and lettuce species were examined, utilizing standard toxicity testing [180–194]. The results indicated that the seedling growths were inversely related to the exposure concentration of Cd, and among the tested plants, the sensitive endpoint appeared in order of tomato, carrot, lettuce, and cucumbers [181–183]. The root growth has not been meaningfully inhibited by the presence of Cd nanomaterials, except for tomatoes, but remarkably promoted by particular Cd nanomaterials [182]. Microscopic images displayed the roots of tested plants exposed to Cd showed a reduction in the root wilt and diameter and the disintegration of the root epidermis; the clutter root surface exhibited evident stress in Cd solution [184–186]. After the addition of Cd nanoparticles, many root hairs and a lack of disintegration on the surface soft of the root system were observed, and Cd nanoparticles crystals were also detected on the plants' root surface [187–189].

#### 3.2.4. TiO_2_: On Plant Growth

Although titanium oxide nanoparticles (TiO_2_) are extensively utilized in daily life products, the research of their uptake and translocation in the plant is restricted, particularly on food crops [195–215]. Due to its small size (<5 nm), TiO_2_ nanoparticles tend to form a covalent bond with most of the no-conjugate natural organic matter, translocate, and following the tissue and cells' specific distribution [198–202]. The overall toxic effects of TiO_2_ nanoparticles are found in the algal species, such as* Desmodesmus subspicatus* [216]. Furthermore, TiO_2_ nanoparticles produce reactive oxygen species upon interaction with organisms or ultraviolet radiation [199–214]. For example, with the presence of TiO_2_, the root of* A. thaliana* releases* mucilage* and forms a pectin hydrogel capsule neighboring the root [205, 208].

**Figure 9:**
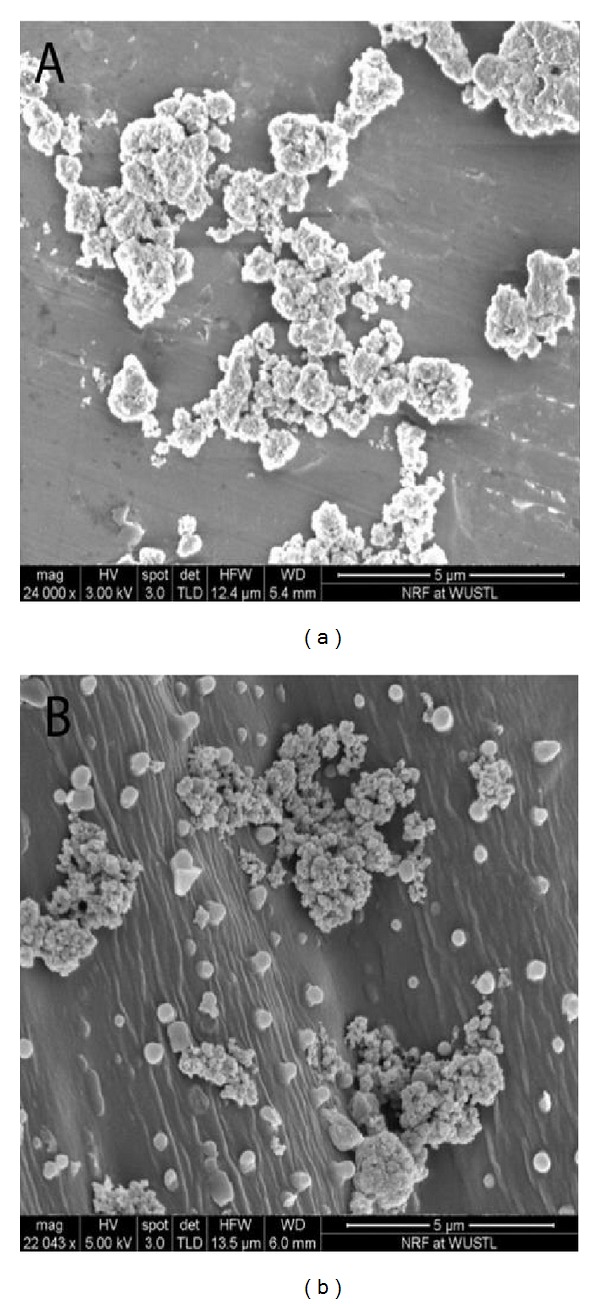
Scanning electron microscope images for NPs/lettuce seeds. In the aqueous phase, the SEM image shows that metal oxide NPs (TiO2 NPs 1000 mgL^−1^) (a) and (CuO NPs 1000 mgL^−1^) were adsorbed on the seed surface (b) [30].

TiO_2_ nanoparticles show an increase in nitrate reeducates in Soybean (*Glycine max*), enhance the ability to absorb/use water, and stimulate the antioxidant system. For example, TiO_2_ nanoparticles treated seeds produced plants that had 73% more dry weight, three times higher photosynthetic rates, and 45% rise in* chlorophyll a* formation compared to the control over the germination period of 30 days [215]. The growth rate of spinach seeds, on the contrary, is proportional to the size of the materials, indicating that the smaller the nanomaterials, the better the germination. Some studies claimed that the TiO_2_ nanoparticles might have elevated the absorption of inorganic nutrients, accelerated the breakdown of organic substances, and caused quenching by oxygen free radicals formed during the photosynthetic process, consequently improving the photosynthetic rate [217, 218]. To increase seed germination rate, the key is the penetration of nanomaterials into the seed [30, 219, 220] (Figure 9). Meanwhile, TiO_2_, in the anatase phase, increases plant growth in spinach by improving nitrogen metabolism that promotes the adsorption of nitrate [30, 218, 219]. The same study indicated the negative effects of TiO_2_ nanoparticles towards the seed germination percentage and the number of roots for the species* Oryza sativa L*. This, in turn, accelerates the conversion of inorganic nitrogen into organic nitrogen, thus increasing the fresh and dry weight [221].

Using bulk and nanosized TiO_2_ at 60 mgL^−1^ promoted sage and seed germination percentages [69, 200]. Exposure of sage seeds to 60 mgL^−1^ bulk and TiO_2_ nanoparticles gained the lowest mean germination time, but higher concentrations did not increase the mean germination time [198, 199]. Application of seeds to TiO_2_ nanoparticles increased the vigor index of sage compared to the control and bulk TiO_2_ treatments.

For spinach seeds, TiO_2_ nanoparticles assisted water absorption, and consequently accelerated seed germination [30, 217]. Thus, some studies declared that altered TiO_2_ nanoparticles were tested in the liquid phase on the plant model* Vicia faba*, which was exposed to three nominal concentrations: 50, 25, and 5 mg commercial sunscreen TiO_2_ nanoparticles per liter for 48 h. Plant growth, photosystem II maximum quantum yield, genotoxicity by micronucleus test, and phytochelatins levels indicated a lack of alteration compared to the control samples. TiO_2_ nanoparticles seem not to exert deleterious effects on our plant model in 48 h, but the observed important clogging onto the roots [222]. It is shown that a combination of nanosized TiO_2 _could improve the nitrate reductase enzyme in soybean (*Glycine max*), increase its abilities of absorbing and utilizing fertilizer and water, encourage its antioxidant system, and actually hasten its germination and growth [223]. In addition, it is stated that the positive effects of TiO_2_ could be due to antimicrobial properties of ENs, which can improve the strength and resistance of plants to stress.

Therefore, the acute toxic effects of TiO_2_ nanoparticles are considered low, with the effects not following a clear dose-effect relationship. This is perhaps due to particle agglomeration and subsequent sedimentation. Genomic DNA quantification was detected in the root tips of cucumber after seven days and indicated that plants treated with 2000–4000 mgL^−1^ of TiO_2_ nanoparticles reduced the genomic DNA compared to the control sample [205, 208]. The toxic effect of TiO_2_ nanoparticles is possibly not attributed by the released Ti^2+^ ions from particles that are tentatively proved by the limited dissolution of Ti from a TiO_2_ sample [200].

However, the presence of TiO_2_ also positively impacts the plants' growth. For example, TiO_2_ nanoparticles were observed to promote the growth of spinach through an improvement in nitrogen metabolism and photosynthetic rate.

#### 3.2.5. Al_2_O_3_: On Plant Growth

Phytotoxicity of uncoated and phenanthrene-coated alumina (Al_2_O_3_) nanoparticles showed that uncoated Al_2_O_3_ nanoparticles at 2 mgL^−1^ concentrations inhibited the root elongation of cucumber, corn, carrot, cabbage, and soybean [224–226]. It is mentioned that the toxic effect is probably not nanospecified but is due to the dissolution of Al_2_O_3_ nanoparticles. The effects of submicron Al_2_O_3_ particles were investigated to evaluate the chemical material that might be toxic towards the growth of seedling roots [225–228]. Thus, particle surface characteristics play a critical role in the phytotoxicity of Al_2_O_3_ nanoparticles [227].

This supported the fact that the presence of Al_2_O_3_ can stunt root growth in cucumber, corn, carrot, cabbage, and soybean, although preliminary findings suggest extremely high concentrations of such particles are necessary to induce damage [229–231]. The presence of Al_2_O_3_ nanoparticles did not have a negative effect on the growth of* Lolium perenne* and* Phaseolus vulgaris* in the tested concentration range [232, 233]. Al_2_O_3_ nanoparticles concentration in rye grass improved the control analysis by 2.5 times, with no uptake was observed in kidney beans, which inferred the difference in the uptake and distribution efficiency of different plants by similar nanoparticles [234–239].

#### 3.2.6. Fe_3_O_4_: On Plant Growth

The excess amount of iron oxide (Fe_3_O_4_) as a magnetic nanomaterial resulted in some negative effect towards plant growth. For example, “Chlorophyll a” levels were amplified at low Fe_3_O_4_ nanoparticles fluid concentrations, while at higher concentrations it inhibited it [240–242]. A small inhibitory effect was discovered on the growth of the plantlets that led to brown spots on leaves at higher volume fractions of Fe_3_O_4_ nanoparticles fluids [243–245]. The excess Fe_3_O_4_ nanoparticles treatment produced some oxidative stress, which in turn affected photosynthesis and resulted in decreased rates of metabolic process [246–248]. The oxidative stress was induced by the Fe_3_O_4_ fluid concentration in the tissues of living plants [245–247].

In order to overcome such limitations, the coating provides Fe_3_O_4_ nanoparticles with a large adsorption surface and biocompatible properties [249–252]. For example, in the case of pumpkin (*Cucurbita pepo*), the presence of carbon coated-Fe_3_O_4_ at certain concentrations within some cells and in extracellular space decreases the problems for plant tissues and the amount of chemicals released into the environment [250]. Furthermore, the influence of tetramethylammonium hydroxide coated Fe_3_O_4_ nanoparticles on the growth of corn (*maize*) found that the chlorophyll level increased at low Fe_3_O_4_ nanoparticle fluid, while at higher concentrations it was inhibited [253].

A slight inhibitory effect was observed in the growth of the plantlets, which in turn resulted in brown spots on leaves at greater volume fractions of the magnetic fluid [254, 255]. The oxidative stress was induced by the Fe_3_O_4_ nanoparticles fluid towards the living plant tissue [256–258]. The excess Fe_3_O_4_ nanoparticles generated some oxidative stress, affected photosynthesis, and resulted in the reduction of metabolic process rates.

#### 3.2.7. Zn/ZnO: On Plant Growth

Zinc (Zn) and zinc oxide (ZnO) are categorized as commonly used metal/metal oxide engineered nanomaterials. Zn is an essential micronutrient for humans, animals, and plants [259–263]. ZnO is mostly utilized in a range of applications such as sunscreens and other personal care products, solar cells, and photocatalysis, biosensors, and electrodes [261]. According to the analysis of 289 soil samples collected from different countries in the world, Zn/ZnO deficiency was found to be the most widespread micronutrient deficiency and the fourth most important yield-limiting nutrient after nitrogen, phosphorus, and potassium [260–262].

Due to its increasing utilization in consumer products, it is quite possible that through both accidental release and deliberate application, Zn/ZnO might find their way into atmospheric environments, whether terrestrial or aquatic [263–266]. It induces noticeable effect on many organisms, especially on plants, which are an essential base component to all ecosystems [267].

**Figure 10:**
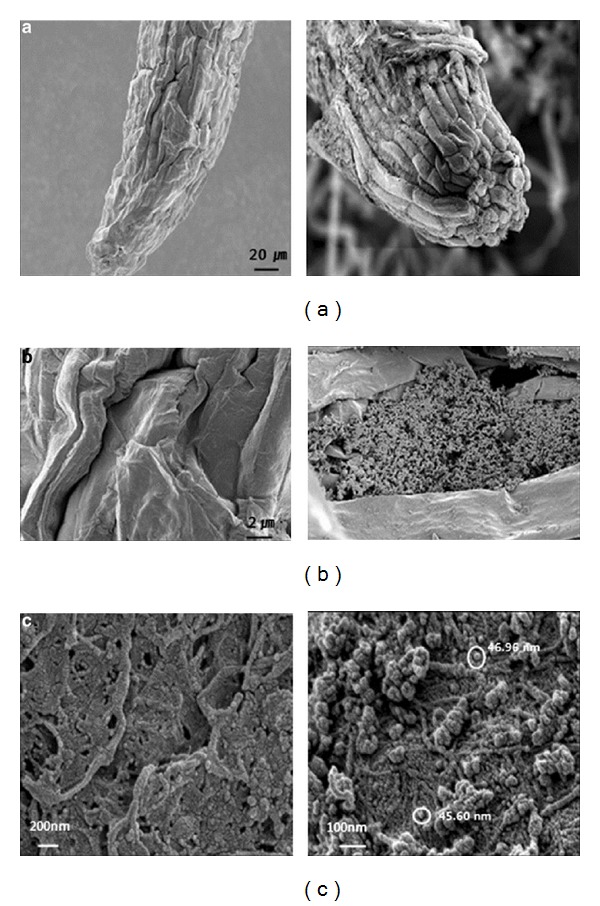
Scanning electron microscope images of Buckwheat (Fagopyrum esculentum) root surface under control (left) and treatment (right) with ZnO nanoparticles (1,000 mgL^−1^) at a magnification of ×1,000 (a), ×5,000 (b), and ×150,000 (c) [31].

**Figure 11:**
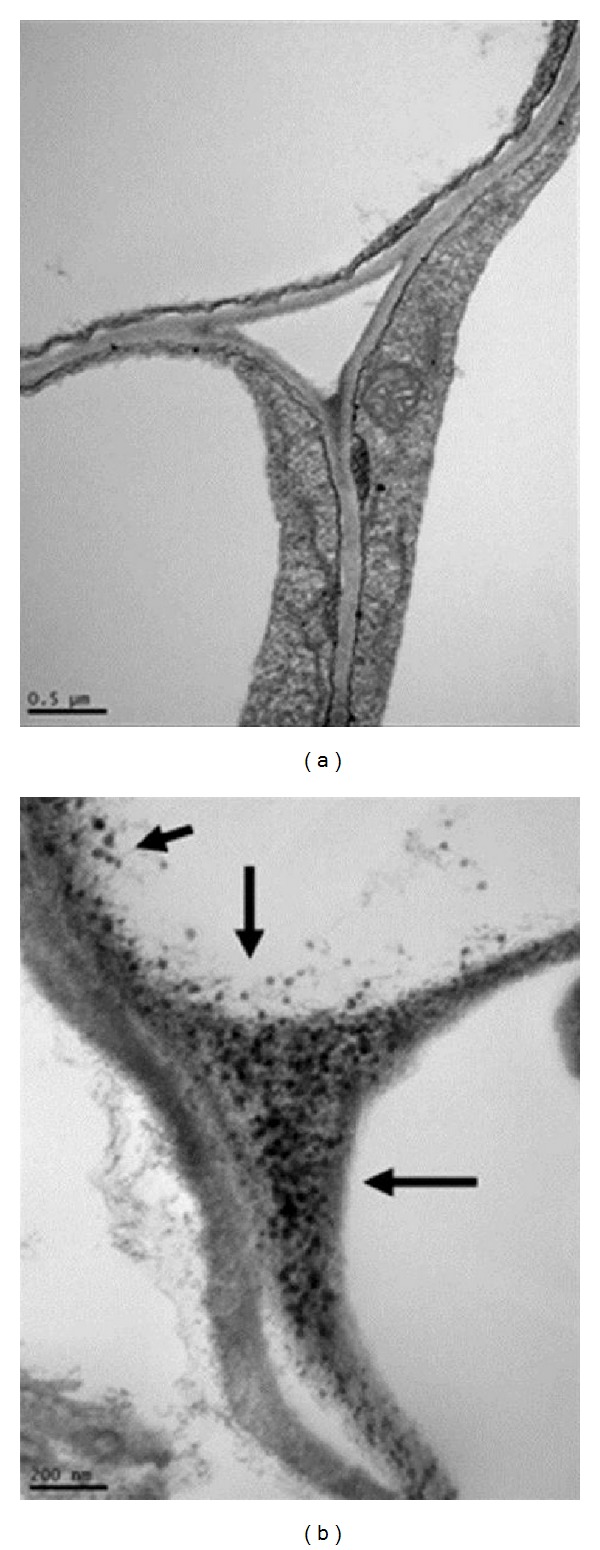
Transmission electron microscopy images of Buckwheat (Fagopyrum esculentum) root surface under control (a) and treatment (b) with ZnO nanoparticles (1,000 mgL^−1^) [31].

A number of researchers described the key role of Zn/ZnO nanomaterials for plant growths and yield [265–267]. For example, higher plant mostly absorbs Zn as a divalent cation (Zn^2+^), which acts either as a functional, structural, or as the metal component of enzymes or a regulatory cofactor of numerous enzymes [266]. Zn nanomaterials are needed for chlorophyll production, fertilization, pollen function, and germination. Among the micronutrients, Zn affects the susceptibility of plants via drought stress [263]. The germination rate of the plant may be affected in the presence of Zn and ZnO. ZnO nanomaterials are hazardous and affect both the chromosomal and the cellular facets. Clear root germination effects, due to the presence of ZnO, were observed for the species of Buckwheat (*Fagopyrum esculentum*) [31] (Figures 10 and 11). Furthermore, the presence of the ZnO nanoparticles also promoted the permeation of onion (*Allium cepa*) roots and effected the roots' elongation, genetic materials, and metabolisms. The ZnO suspension meaningfully inhibited root growth of corn, with the termination of root development.

Growth of roots was halted with seed soaking and incubation in the suspension of Zn/ZnO nanoparticles. It also indicates that Zn^2+^ is more toxic in ryegrass species compared to ZnO nanoparticles [31, 258–268]. The root growths are found in seedling of ryegrass, radish, and rape exposed to less than 10 mgL^−1^ of ZnO/Zn nanoparticles. The toxicity of ZnO nanoparticle could not mostly come from its dissolution at the root surface, but also inside the tissue [31].

The toxicity of ZnO nanoparticle and Zn^2+^ could be derived by two theories; a chemical toxicity based on chemical composition and the stress or stimuli caused by the size, shape, and surface of the ZnO nanoparticles [266]. Both theories significantly affected the cell culture response of the plants. A number of mechanisms underlined the efficiency of Zn/ZnO. Depending on the plant species and the experimental conditions, the most important mechanism may be Zn/ZnO utilization in tissues, called the internal efficiency, or Zn/Zn uptake, called the external efficiency [267]. This, in turn, helped ZnO nanoparticle enter the root cells and inhibit seedling growth.

#### 3.2.8. Cu/Cu_2_O: On Plant Growth

Copper/Copper oxide (Cu/Cu_2_O) nanoparticles could block water channels through adsorption and increase the possibility for radical penetration into onion roots [269–271]. This, in turn, spoils the complete stages of cell division and cellular metabolism [270]. The bioavailability and toxicity of Cu nanoparticles to the Mung bean (*Phaseolus radiates*) and wheat (*Triticum aestivum*) species employed plant agar test as a growth substrate for the homogenous exposure of nanoparticles. The rate of growth for both species were inhibited; as a result of exposure to Cu, nanoparticles and the seedling length of tested species are inversely related to the exposure concentration of Cu nanoparticles.

The toxicity and bioavailability of Cu nanoparticles were observed on the plants Mung bean (*Phaseolus radiates*) and wheat (*Triticum aestivum*). The observation employed plant agar test as the growth substrate for the homogeneous exposure of nanoparticles [272–274]. The growth rates of both plants were inhibited, and due to the exposure to nanoparticles and the seedlings, the lengths of the tested species were inversely related to the exposure concentration of the nanoparticles [273]. Wheat crop showed a greater accumulation of Cu nanoparticles in its roots due to the roots' morphology. The bioavailability was estimated by calculating the bioaccumulation factor defined as the Cu nanoparticles concentration in the plants, divided by the concentration of Cu nanomaterials in the growth media [275]. Growth inhibition of a seedling exposed to different concentrations of Cu nanomaterials on Mung bean (*Phaseolus radiates*) was more sensitive compared to wheat (*Triticum aestivum*) [276]. A cupric ion released from Cu nanoparticles had negligible effects on the concentration ranges of the present study, and the apparent toxicity was clearly the result of Cu nanomaterials [277, 278].

With increasing concentration of Cu nanoparticles and agglomeration of particles, the rates of bioaccumulation increased. Bioaccumulation of Cu nanoparticles increased with its concentration in the growth media, and their bioavailability to the test plants was estimated by calculating the bioaccumulation factor. Some studies demonstrated the plant agar test as a good protocol to test phytotoxicity of Cu nanoparticles, which is hardly water-soluble [279]. Moreover, studies on the effects of Cu nanoparticles on the growth of zucchini plants showed the reduced length of emerging roots [275], although the germination of lettuce seeds in the presence of Cu nanomaterials showed an increase in the shoot-to-root ratio compared to the control plants [280].

Bioavailability was estimated by calculating the bioaccumulation factor defined as the Cu nanoparticles concentration in the plants divided in the growth media by its concentration. Growth inhibition of a seedling exposed to different concentration of Cu nanomaterials on Mung bean (*Phaseolus radiates*) was more sensitive than wheat (*Triticum aestivum*). Cu^2+^ ions released from Cu nanomaterials had negligible effects on the concentration ranges and the apparent toxicity clearly resulting from Cu nanoparticles [276]. Thus, bioaccumulation increased with the concentration of Cu nanoparticles and the agglomeration of the particles [281]. The increments on bioaccumulation were derived from growth media and its bioavailability to the tested plant was estimated by calculating the bioaccumulation factors. Studies on the effect of Cu nanoparticles on the growth of zucchini plants indicated a reduction in the length of emerging roots [282]. However, the germination of lettuce seeds in the presence of Cu nanomaterials showed an increment in the shoot-to-root ratio compared to the control plant. The effect of Cu nanoparticles' toxicity to the plant and food crops is evident, with the clear impacts on crop growth, root length, shoot length, biomass accumulation, and germination visualized from the contaminated plants [281].

#### 3.2.9. Ce/CeO_2_: On Plant Growth

Cerium oxide (CeO_2_) nanoparticles utilized in several emerging applications which leverage the UV absorbing capacity and high O_2_ storage of CeO_2_ nanoparticle and the low redox potential of the Ce IV/Ce III redox couple [35]. The natural environment may expose to CeO_2_ nanoparticle from exhaust catalysts after deposition on plant, when they are collected with road runoff, or by industrial wastewaters that contain CeO_2_ nanoparticles. Very fine (<1 *μ*m) exhaust particulates cause very diffuse pollution, and CeO_2_ nanoparticles contamination does not cause a noteworthy cerium (Ce) enrichment in natural waters. Besides, CeO_2_ nanoparticles, as the only tetravalent metal oxide, showed very different effects on the test plant species [35].

Possible toxicity, transport, fate and of CeO_2_ nanoparticles remain unknown. Some works have focused on effect of CeO_2_ (concentration at 0 to 4000 mgL^−1^) on seeds of tomato (*Lycopersicon esculentum*), cucumber (*Cucumis sativus*), alfalfa (*Medicago sativa*), and corn (*Zea mays*). The results found that CeO_2_ nanoparticles meaningfully decreased corn germination (about 30% at 2000 mgL^−1^; *P* < 0.05), and at 2000 mgL^−1^, the germination of tomato and cucumber was reduced by 30 and 20%, respectively (*P* < 0.05) [283]. The root growth significantly promoted (*P* < 0.05) by CeO_2_ nanoparticle in cucumber and corn but reduced (*P* < 0.05) in alfalfa and tomato. However, a suspension of 2000 mgL^−1^ CeO_2_ nanoparticle had no effect on the root elongation of radish, rape, tomato, wheat, cabbage, and cucumber, except lettuce [257].

Furthermore, few reports consequently far have addressed the entire life cycle of plants grown in ENs-contaminated soil. Soybean (*Glycine max*) seeds germinated and grown to full maturity in organic farm soil amended with either ZnO ENs at 500 mg/kg or CeO_2_ ENs at 1000 mg/kg. In other study, the short-term effects of CeO_2_ nanoparticle in two different agglomeration states on the green algae* Chlamydomonas reinhardtii* were examined. It demonstrated that the level of dissolved cerium (III) in CeO_2_ ENs suspensions was very low and between 0.1 and 27 nM [284]. The agglomerated CeO_2_ ENs caused a slight decrease of photosynthetic yield at the highest concentrations (100 M), while no effect observed for dispersed CeO_2_ ENs. The low toxicity of agglomerated CeO_2_ ENs was attributed quantitatively to Ce^3+^ ions cooccurring in the nanoparticle suspension whereas for dispersed CeO_2_ ENs, dissolved Ce^3+^ precipitated with phosphate and not bioavailable [257, 283].

## 4. Engineered Nanomaterials: Interaction and Mechanism in Plant

Interaction of ENs and plants can be categorized under phytotoxicity, uptake, translocation, and accumulation. Current literature revealed that all of the aforementioned interactions depend on the species of the plant, its type, size, chemical composition, stability, and functionalization of ENs.

### 4.1. Engineered Nanomaterials: Phytotoxicity Mechanism

Phytotoxicity studies using higher plants are an important criterion for understanding the toxicity of ENs. The vast majority of research dedicated to the potential toxicity of ENs to plants and both negative and positive or inconsequential effects have been reported [48, 52, 97]. The majority of the reports available in the literature indicate the phytotoxicity of ENs [94, 104]. For example, a pronounced increase in the rate of germination was observed for rice seeds in the presence of some of carbon nanomaterials, particularly CNTs [101–103]. Increased water content observed in the CNT-treated seeds during germination was compared to the control samples. The germinated seeds grown in a basal growth medium were supplemented with CNTs in order to study their impact on further seedling growth [102]. The results indicate possible use for CNTs as enhancers in the growth of rice seedlings. Another example derived from Al_2_O_3_ nanomaterials inhibit root elongation of cucumber, corn, soybeans, carrot, and cabbage [225–228], while ZnO nanomaterials were reported to be one of the most toxic nanomaterials that could terminate root growth of test plants [260–262]. Similar studies were carried out on the toxicology of Al_2_O_3_, SiO_2_, ZnO, and Fe_3_O_4 _on* Arabidopsis thaliana*, with the results showing that ZnO nanomaterials at 400 mgL^−1^ capable of inhibiting germination [265–267]. From a toxicological perspective, surface area and particle size are important material characteristics. As the size of the particles decreases, its surface area increases, and allows a greater proportion of its atoms or molecules to be displayed on the surface rather than the interior of the ENs [28, 117]. The increase of surface area determines the potential number of reactive groups on the particles' surface [117]. The change in the structural and physicochemical properties of ENs, with a decrease in size, could be responsible for a number of material interactions that could result in toxicological effects [178]. One of the earliest observations on the effect of surface properties on toxicity of ENs showed greater toxicity than fine particulate of similar materials on the basis of mass [182, 183]. This has been observed with different kinds of ENs, including TiO_2_, carbon black, Co, and Ni. It was found that TiO_2_ nanoparticle with a size of 21 nm resulted in 43 times more inflammation than 250 nm particles based on similar mass [198–202]. The increase in inflammation is believed to be caused by much greater surface area of the small particles for similar masses of material. Another example derived from the applicability of fluorescein isothiocyanate labeled silica nanoparticles and photo-stable Cadmium-Selenide quantum dots were tested for their ability to be used as biolabels, and for promoting seed germination [285–287]. It was found that FTIC-labeled silica nanoparticles induced seed germination in rice, while quantum dots arrested the germination. Multiple studies showed that nanosized particles are more toxic than microsized particles [286]. Intrinsic surface reactivity is another factor that determined the toxicity of ENs. It was determined that other types of crystalline anatase TiO_2_ did not show size intensive toxicity for nanosized particles [30, 219, 220]. Overall, the current phytotoxicity profile of ENs is highly speculative and preliminary, and the effects of their unique characteristics are poorly understood and more studies on toxicity are required, especially on commercial food crop [185].

Toxic effect of ENs brought from the dissolved species that originated from dissolution, which in turn could increase the damage to genetic materials, agglomeration, and biomass production, while reducing the length of the roots [117, 183]. However, there are still positive effects on the accumulation of nanomaterials in plants, especially in multiwall carbon nanotube, Zn, and ZnO [265–267]. The presence of these nanomaterials induces the germination and seedling growth of* Brassica Juncea* and black gram (*Phaseolus Mungo*) [94]. The toxicity of various organisms depends on the nature of particles, sizes, concentration, and exposure times [186, 187]. The theory on the extension effect remains unclear, from nanoformulation, fraction, or size of the nanomaterials [193, 199]. Nevertheless, some studies have indicated that the phytoxicity observed on the exposure to ZnO nanoparticles may be attributed solely to dissolved-Zn, which was similar to the conclusion drawn regarding Au nanoparticles [259]. Another study discovered that the toxic effect by ZnO is more significant in seed germinations, root elongations, and the number of leaves, rather than other nanoparticles [263]. Furthermore, studies on the relevance of phytotoxicity on rice (*Oryza sativa*) towards Au nanoparticles have been analyzed and from the micrograph analysis found that various particle sizes deposited inside the root cells through the small pores of cell walls are done via cellular mechanism [136–138].

In conclusion, most of the studies demonstrated direct exposure to specific types of nanoparticles causing significant phytotoxicity, emphasizing the need for ecologically responsible disposal of wastes containing ENs. It also highlights the necessity for further study on the impacts of ENs on agricultural and environment systems.

### 4.2. Engineered Nanomaterials: Uptake Mechanism

One of the major research gaps on the uptake mechanism of nanomaterials towards plants is the absence of consistent and broadly applicable information [65, 278]. Most information revealed that ENs could adhere to plant roots and exert chemical or physical uptakes upon plants [245, 273]. Recently, there are an increasing number of publications emerging on the interaction of ENs with plants [241]. The uptake, accumulation, and build-up of nanoparticles vary, and these factors largely depend on the type, size, and the composition of the plant. Indeed, the verification on the uptake mechanism of ENs is limited and is focused on stock solutions rather than the actual concentration [102, 139]. The stock solution is prepared either from a series of dilution or media renewable periods. As such, most method being reported might not produce similar results for different shapes, sizes, and forms of nanomaterials [187]. Most of the data correspond to the germination stage and cell culture, which are mostly focused on metal-based nanomaterials, such as TiO_2_, CeO_2_, Fe_3_O_4_, ZnO, Au, Ag, Cu, and Fe. In this case, only fullerene and fullerols showed a ready uptake in plants.

Several avenues for the uptake of nanomaterials by plant cells are proposed. Some of the data suggested that the nanomaterials could enter plant cells by being bound to a carrier protein, through aquaporin, ion channels, or endocytosis via the creation of new pores, ending up being bounded to organic chemicals [191, 205]. This phenomenon is preferred in the case of carbon nanotubes rather than other types of nanomaterials [102]. Meanwhile, the greater surface area-to-mass ratio of the nanoparticle compared to the bulk metals induces higher reactivities compared to the surroundings [213]. Consequently, the nanomaterials may form complexes with membrane transporters or root exudates before being transported into the plants. Most metal-based nanomaterials that have been reported as being taken up by plants include elements for which ion transporters have been identified [278]. Once the nanomaterials enter the plant cells, it may be transported either apoplastically or symplastically from one cell to another via plasmodesmata [139].

However, the relations between the selectivity of the uptake of nanomaterial and the type of plant remain unknown and are open to exploration. Some studies suggested that the gradual increase in ENs uptake was observed with reducing granule size, and only the powder from produced plants with ENs concentrations remains in the sufficient range [213, 273]. For example, ZnO granule of 1.5 mm weigh less than granules of 2.0 or 2.5 mm, smaller granules utilized for similar weights, resulting in a better distribution of Zn and the higher surface contact area Zn fertilizer, resulting in better Zn uptake [266]. Ample work was done, emphasizing the role of particle size in increasing the efficiency of ENs uptake and higher yields.

### 4.3. Engineered Nanomaterials: Translocation Mechanism

Prior to translocation, engineered nanomaterials are intermediate in its mobility or phloem export. Some studies suggested that the translocation of ENs depends on the amount being supplied and the nature of the plant as a species [11]. Engineered nanomaterials move from leaves to roots, stem, and developing grain, and from one root to another. The higher translocation of other nutrient is recorded by the increment on its demand [240]. The translocation mechanism is initiated with the penetration of ENs through cell walls and plasma membrane of root cells. One of the main passages of uptake and transportations to the shoot and leave(s) of plant is the Xylem [288, 289]. In this case, the pore size of cell wall must be in range of 3–8 nm, which is smaller than ENs. The penetration rate was studied with leek (*Allium porrum*), and it was found that ENs pathway in the leaf was followed with the stomatal pathway [289].

### 4.4. Engineered Nanomaterials: Transmission Mechanism

The first step to understand the possible benefits of applying nanotechnology to agriculture should be to analyze the transmission mechanism of ENs in plants. Transmission of ENs was detected at different levels: chains of nanomaterials-aggregates carrying cells apparently close to the application point, when such application was made by the “injection” of the ENs suspension into the pith cavity of the stem, suggesting the presence of flux of nanoparticles from one cell to another [290]. The nanomaterials are capable of penetrating through the leaf cuticle and into the cell cytoplasm [291, 292]. Plants provide a potential pathway for the transport of nanomaterials to the environment and serve as an important route for their bioaccumulation into the food chain [292]. The wall of the plant's cell acts as a barrier for easy entry to any external agents, including ENs into plant cells. The sieving properties are determined by the pore diameter of the cell wall, ranging from 5 to 20 nm [290]. Only ENs aggregates with diameters less than the pore diameter of the cell wall could easily pass through and reach the plasma membrane [291].

There is also a chance for the enlargement of pores or induction of new cell wall pores upon interaction with ENs, which will in turn enhance nanoparticle transmissions [293]. They may also cross the membrane using embedded transport carrier proteins or through ion channels. In the cytoplasm, the ENs may bind with different cytoplasmic organelles and interfere with the metabolic processes at that site [291].

When ENs is applied on the surface of leaves, they will enter through the stomata openings or through the bases of trichomes and then translocated to various tissues. However, the accumulation of ENs on photosynthetic surface causes foliar heating, which results in the alterations to gas exchange, due to stomata obstruction that produces changes in various physiological and cellular functions of plants. The application of microscopy techniques visualizes and tracks the transport and deposition of ENs inside the plants [294]. The ENs tagged to agrochemicals or to other substances could reduce the injury to plant tissues and the amount of chemicals released into the environment; a certain contact is however unavoidable, due to the strong interaction of plants with soil growth substrates [295].

This limitation is circumvented by coating. For example, the carbon-coated Fe_3_O_4_ nanomaterials (carbon encapsulation provides biocompatibility and a large adsorption surface) in living plants such as pumpkins (*Cucurbita pepo*) and the results showed the presence of nanomaterials both in the extracellular space and within some cells [245].

One of the pathways also reported particle size of 20 nm Ag nanoparticles may be transported inside the cells through plasmodesmata [169–171]. Particles must enter through the cell wall and the plasma membrane of root cells. Xylem is one of the main passages of uptake and transportations to the shoot and the leaves of plant. Pore size of cell wall was in the range of 3–8 nm, which is smaller than ENs. The penetration rates of foliar applied to polar solutes are highly variable and the mechanism is not fully understood [294]. Investigation in leek (*Allium porrum*) and broad bean (*Vicia faba*) size exclusion limits and lateral heterogeneity of the stomata foliar uptake pathway for aqueous solutes and water-suspended nanoparticles were done in [148, 176, 177]. Thus, the nanomaterials pathway in leaf follows the stomata pathway, which differs fundamentally from the cuticolar foliar uptake pathway [269]. This consequently proved the limitation of transmission and the distribution of Ag nanoparticles in* Medicago sativa* and* Brassica juncea*. In contrast to* Brassica juncea*,* Medicago sativa* showed an increase in metal uptakes with a corresponding increase in the substrate of metal concentration and exposure time [146]. The Ag nanoparticles were located in the nucleus and applied the definition that suggested both* Brassica juncea* and* Medicago sativa* hyperaccumulators of Ag nanoparticles.

## 5. Conclusion and Prospective

This work proves that certain engineered nanomaterials could exert chemical or physical toxicity on plants depending on its size, chemical composition, surface energy, and species, leading to different techniques. Hence, the challenges for further research is the uptake kinetics and the interaction mechanism within cells, and also the maximum agreeable amount of these engineered nanomaterials that plants can take without showing any signs of stress. An extensive research on the toxic effects of nanomaterials could meaningfully help by utilizing and disposing engineered nanomaterials for the reduction of adverse effects in both of agricultural and of environmental systems.

## Figures and Tables

**Table 1:** Classification of nanomaterials.

Categories of nanomaterials	Description	References
Nanoparticles	Submicron or even ultramicron size particles obtainable as high performance radiant resistant materials, magnetic materials, solar battery materials, packaging materials, and magnetic fluid materials	[11, 12]

Nanotubes and nanofibers	Nanometer size long linear material, optical materials, micro conductors, microfibers, nanotubes of PEEK, PET, and PTFE	[8]

Nanofilm	Films utilized as gas catalyst materials	[18]

Nanoblock	Nanometer crystalline materials produced by substantial accuracy, developing controlled crystallization or nanoparticles	[19]

Nanocomposites	Composite nanomaterials, which use nanosize reinforcements instead of conventional fibers or particulates	[15]

Nanocrystalline solids	Polycrystals with the size of 1 to 10 nm and 50% or more of solid consists of inherent interface between crystals and different orientations. The clusters that formed through homogenous nucleation and grow by coalescence and incorporation of atoms.	[16]
